# A Close Approach to a Correct Conception of Malaria in 1684

**Published:** 1915-03

**Authors:** 


### HISTORIC NOTE.

### A Close Approach to a Correct Conception of Malaria in 1684.

La Hontan writes as follows, from Montreal, Nov. 2, 1684: Toward the end of August, M. de la Barre joined us, but he was dangerously ill of a fever, which raged in like manner among most of his militia, so that only our three companies were free from sickness. This fever was of the intermitting kind, and the convulsive motions, tremblings and frequency of the pulse that attended the cold fit, were so violent, that most of our sick men died in the second or third fit. Their blood was of a blackish brown color and tainted with a sort of yellowish serum, not unlike pus or corrupt matter. M. de la Barre's physician, who, in my opinion knew as little of the true causes of fevers as Hippocrates or Galen, and a hundred thousand besides; this mighty physician, pretending to trace the cause of the fever, imputed it to the unfavorable qualities of the air and the aliment. His plea was that the excessive heat of the season put the vapors or exhalations into an over-rapid motion; that the air was so over-rarified that we did not suck in a sufficient quantity of it, that the small quantity that we did receive was loaded with insects and impure corpusculums, which the fatal necessity of respiration obliged us to swallow and that by this means nature was put out of order. He added that the use of brandy and salt meat soured the blood, that this sourness caused a sort of coagulation of the chyle and blood, that the coagulation hindered it to circulate through the heart with a due degree of celerity, and that thereupon there ensued an extraordinary fermentation which is nothing else but a fever. La Hontan ascribes the relative immunity of his companies and of the Indians to exposure of de la Barres's troops in poling their canoes through cold water. Considering the ubiquitous mosquitoes and gnats that he mentions and his contempt for the doctor, it is quite possible that the latter not only had a pretty fair conception of the action of germs, acidosis and infection resulting from lack of resistance to extrinsic causes but that he may even have guessed how the malarial parasite entered the system.

